# Sex difference in physical activity, energy expenditure and obesity driven by a subpopulation of hypothalamic POMC neurons

**DOI:** 10.1016/j.molmet.2016.01.005

**Published:** 2016-01-22

**Authors:** Luke K. Burke, Barbora Doslikova, Giuseppe D'Agostino, Megan Greenwald-Yarnell, Teodora Georgescu, Raffaella Chianese, Pablo B. Martinez de Morentin, Emmanuel Ogunnowo-Bada, Celine Cansell, Lourdes Valencia-Torres, Alastair S. Garfield, John Apergis-Schoute, Daniel D. Lam, John R. Speakman, Marcelo Rubinstein, Malcolm J. Low, Justin J. Rochford, Martin G. Myers, Mark L. Evans, Lora K. Heisler

**Affiliations:** 1 Rowett Institute of Nutrition and Health, University of Aberdeen, Aberdeen, UK; 2 Department of Medicine and Institute of Metabolic Science, University of Cambridge, Wellcome Trust/Medical Research Council, Cambridge, UK; 3 Department of Pharmacology, University of Cambridge, Cambridge, UK; 4 Division of Metabolism, Endocrinology, and Diabetes, Department of Internal Medicine, University of Michigan, Ann Arbor, MI, USA; 5 Department of Molecular and Integrative Physiology, University of Michigan Medical School, Ann Arbor, MI, USA; 6 Institute of Biological and Environmental Sciences, University of Aberdeen, Aberdeen, UK; 7 State Key Laboratory of Molecular Developmental Biology, Institute of Genetics and Developmental Biology, Chinese Academy of Sciences, Beijing, China; 8 Instituto de Investigaciones en Ingeniería Genética y Biología Molecular, Consejo Nacional de Investigaciones Científicas y Técnicas, 1428, Buenos Aires, Argentina

**Keywords:** *Pro-opiomelanocortin (Pomc)*, 5-HT2c receptor, Obesity, Energy expenditure, Brown adipose tissue, Hyperinsulinemia, Sexual dimorphism, Hypothalamus

## Abstract

**Objective:**

Obesity is one of the primary healthcare challenges of the 21st century. Signals relaying information regarding energy needs are integrated within the brain to influence body weight. Central among these integration nodes are the brain pro-opiomelanocortin (POMC) peptides, perturbations of which disrupt energy balance and promote severe obesity. However, POMC neurons are neurochemically diverse and the crucial source of POMC peptides that regulate energy homeostasis and body weight remains to be fully clarified.

**Methods:**

Given that a 5-hydroxytryptamine 2c receptor (5-HT_2C_R) agonist is a current obesity medication and 5-HT_2C_R agonist's effects on appetite are primarily mediated via POMC neurons, we hypothesized that a critical source of POMC regulating food intake and body weight is specifically synthesized in cells containing 5-HT_2C_Rs. To exclusively manipulate *Pomc* synthesis only within 5-HT_2C_R containing cells, we generated a novel *5-HT*_*2C*_*R*^*CRE*^ mouse line and intercrossed it with Cre recombinase-dependent and hypothalamic specific reactivatable *Pomc*^*NEO*^ mice to restrict *Pomc* synthesis to the subset of hypothalamic cells containing 5-HT_2C_Rs. This provided a means to clarify the specific contribution of a defined subgroup of POMC peptides in energy balance and body weight.

**Results:**

Here we transform genetically programed obese and hyperinsulinemic male mice lacking hypothalamic *Pomc* with increased appetite, reduced physical activity and compromised brown adipose tissue (BAT) into lean, healthy mice via targeted restoration of *Pomc* function only within 5-HT_2C_R expressing cells. Remarkably, the same metabolic transformation does not occur in females, who despite corrected feeding behavior and normalized insulin levels remain physically inactive, have lower energy expenditure, compromised BAT and develop obesity.

**Conclusions:**

These data provide support for the functional heterogeneity of hypothalamic POMC neurons, revealing that *Pomc* expression within 5-HT_2C_R expressing neurons is sufficient to regulate energy intake and insulin sensitivity in male and female mice. However, an unexpected sex difference in the function of this subset of POMC neurons was identified with regard to energy expenditure. We reveal that a large sex difference in physical activity, energy expenditure and the development of obesity is driven by this subpopulation, which constitutes approximately 40% of all POMC neurons in the hypothalamic arcuate nucleus. This may have broad implications for strategies utilized to combat obesity, which at present largely ignore the sex of the obese individual.

## Highlights

• Generation of *5-HT_2C_R^YFP^* mouse line revealed 40% of hypothalamic POMC neurons co-express 5-HT_2C_Rs in male and female mice.
• *Pomc* expressed exclusively within 5-HT_2C_R cells modulates energy intake and insulin sensitivity in male and female mice.
• An unexpected sex difference in physical activity and energy expenditure is modulated by hypothalamic *Pomc*.
• *Pomc* expressed exclusively within 5-HT_2C_R cells differentially impacts body weight accumulation in male and female mice.

## Graphical abstract

**Figure:**
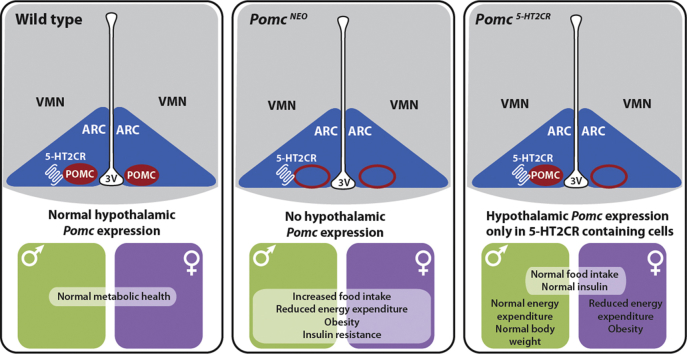

## 1 Introduction

When energy intake exceeds energetic demands, energy is stored primarily as fat. Excess adipose accumulation is the hallmark feature of obesity. Though men and women are exposed to the same environmental conditions, the World Health Organization (WHO) reports higher rates of obesity in women worldwide, reaching twice the prevalence of men in some regions of the world [1]. Obesity has a significant and widespread impact on human health, placing it at the forefront of healthcare priorities and challenges of this century. Obesity medications are in general prescribed without attention to the sex of the obese individual, implying the absence of sex-based differences in the molecular regulation of energy balance.

Insights from genetic research have led to the discovery of key regulators of energy balance [2], such as the melanocortin peptides encoded by the pro-opiomelanocortin gene (*Pomc*) [3]. Humans and animals unable to synthesize melanocortins or the receptor through which they primarily signal to influence energy balance, the melanocortin4 receptor (*MC4R/Mc4r*), have dramatically increased food intake, reduced physical activity or energy expenditure and develop profound obesity [4], [5]. *POMC/Pomc* function also has broader application to common obesity; in both high fat diet-induced obesity and middle-age associated obesity, POMC neuron activity within the arcuate nucleus of the hypothalamus (ARC) is diminished, which has been proposed to have a causal role in the increased acquisition of body weight and adiposity [6], [7], [8]. Treatment with a 5-hydroxytryptamine 2c receptor (5-HT_2C_R) agonist, such as the new obesity medication lorcaserin (Arena Pharmaceuticals), restores diminished POMC neuron function and improves obesity [9], [10], [11]. Furthermore, inactivating 5-HT_2C_Rs specifically on POMC neurons in mice, a genetic strategy employed to manipulate 5-HT_2C_R expression, prevents the anorectic effect of 5-HT_2C_R agonists [12], thereby revealing that 5-HT_2C_R agonists modulate food intake via POMC neurons. Thus, POMC peptides are an important driver of body weight and POMC expressing neurons are amenable to pharmacological manipulation. Here, we sought to clarify the source of POMC peptides that critically mediate body weight using a newly developed genetic approach.

## 2 Materials and methods

### 2.1 Mice

*5-HT*_*2C*_*R*^*CRE*^ line. 5.6 kb of genomic DNA containing portions of the final exon and the 3′ UTR of the murine *Htr2c* gene was amplified by PCR from R1 ES cells [(129X1/SvJ × 129S1)F1 genetic background] and cloned into a plasmid for insertion of a FRT-NEO-FRT-IRES-CRE cassette between the STOP codon and the polyadenylation site, as previously described [17]. The targeting construct was linearized using NotI and electroporated into R1 mouse embryonic stem cells at the University of Michigan Transgenic Animal Model Core. Neomycin-resistant clones were analyzed by quantitative real-time PCR [18] for copy number of the native *Htr2c* allele and further confirmed by Southern blotting using an external probe. Correctly targeted ES cells were injected into C57BL/6J blastocysts to generate chimeras. Male chimeras were then bred to C57BL/6J females, and pups were genotyped to confirm insertion of IRES-Cre into the appropriate locus. These *5-HT*_*2C*_*R*^*CRE*^ pups were then bred to a germline FlpO deleter strain (129S4/SvJae-Gt(ROSA)26Sortm2(FLP*)Sor/J; Jackson Laboratory) to remove the Neo cassette. Pups positive for FlpO and *5-HT*_*2C*_*R*^*CRE*^ were genotyped for loss of the neo cassette in *5-HT*_*2C*_*R*^*CRE*^ and further bred away from the FlpO allele.

*5-HT*_*2C*_*R*^*CRE*^ mice were then intercrossed with either ROSA26-stop-enhanced yellow fluorescent protein (YFP) (B6.129X1-*Gt(ROSA)26Sortm1(EYFP)Cos*/J; Jackson Laboratory) to create a reporter *5-HT*_*2C*_*R*^*YFP*^ line or *Pomc*^*NEO*^ mice [13] to generate wild type, *5-HT*_*2C*_*R*^*CRE*^, ARC *Pomc* null (*Pomc*^*NEO*^), and restored *Pomc* specifically in 5-HT_2C_R expressing cells (*Pomc*^*5-HT2CR*^) littermates.

All mice were group housed and maintained on a 12 h light/dark cycle with *ad libitum* access to water and standard laboratory chow diet. All experiments were in accordance with guidelines and approvals of the University of Michigan or the U.K. Animals (Scientific Procedures) Act 1986.

### 2.2 Immunohistochemistry (IHC)

Tissue was processed for endogenous POMC and for 5-HT_2C_R^YFP^ as previously described [9], [10], [11]. Briefly, under deep terminal anesthesia, mice were transcardially perfused with phosphate buffered saline (PBS) followed by 10% neutral buffered formalin (Sigma). Brains were extracted, post-fixed in 10% neutral buffered formalin at 4 °C, cryoprotected in 20% sucrose at 4 °C and then sectioned coronally on a freezing sliding microtome at 25 μm. Tissue was processed for POMC-immunoreactivity (IR) and 5-HT_2C_R^YFP^ (GFP-IR) as previously described [14], [15] using rabbit anti-POMC primary antibody (1:1000; H-029-30, Phoenix Pharmaceuticals, Burlingame, CA, USA), chicken anti-GFP (1:500; ab13970, AbCam, Cambridge, UK) and Alexa Fluor secondary antibodies (1:500 A-11012, Life Technologies, Paisley, UK), respectively. Single and dual-labeled POMC-IR and GFP-IR cells were counted in the ARC [16]. Analysis was carried out on 7 levels of ARC (−1.46 to −2.18 from Bregma) for each mouse (n = 4/sex).

### 2.3 Quantitative PCR

Total RNA was purified from whole hypothalamus, brainstem and interscapular brown adipose tissue (BAT) using RNA STAT 60 (AMS Biotechnology, Abington, UK) according to the manufacturer's instructions and as previously described (9 months of age; n = 5–9/genotype/sex) [13]. cDNA was obtained by reverse transcription of 500 ng hypothalamic RNA, 1000 ng brainstem RNA and 500 ng BAT RNA. Real-time PCR analysis of cDNA was performed in duplicate on an ABI Prism 7900 sequence detection system using Taqman or Sybr assays for *Pomc* (ABI Taqman Gene expression assay Mm00435874_m1), *elongation of very long fatty acids-like 3* (*Elovl3*) and *peroxisome proliferator-activated receptor gamma coactivator-1alpha (Pgc-1a*). Data for levels of target gene mRNAs are expressed in arbitrary units corrected to the geometric average of four housekeeping genes: *18s*, *36β4*, *βactin* and *glyceraldehyde-3-phosphate dehydrogenase (Gapdh)*. Sequences of primers and probes used are listed in Supplementary Table 1.

### 2.4 Metabolic profile

Body weight was measured from weaning up to 1 year of age (n = 7–17/genotype/sex). Home cage 24-h food intake was measured up to 6 months of age (n = 5–9/genotype/sex). At 9 months of age, a more detailed energy balance profile was performed, including light and dark cycle food intake, locomotor activity and energy expenditure assessment using indirect calorimetry in a Metabolic-Trace (Meta-Trace) system (Ideas Studio, UK; n = 5–9/genotype/sex). Body composition was also analyzed at 7–9 months of age using dual-energy X-ray absorptiometry (DEXA) Lunar PIXImus2 mouse densitometer (General Electric Medical Systems, Fitchburg, WI, USA; n = 5–11/genotype/sex).

Gonadal white adipose tissue (WAT) and interscapular BAT was dissected, fixed in 10% neutral buffered formalin, embedded in paraffin, cut into 5 μm sections and stained with hematoxylin and eosin. WAT cell diameter (μm) and BAT lipid droplet size (% of total area) was measured on an inverted light microscope (Olympus BX41, Olympus UK Ltd, Southend-on-Sea, UK) using CellˆD Olympus Software (Shinjuku, Tokyo, Japan). Analysis was carried out on 5-9 sections for each mouse (9 months of age; n = 4–6/genotype/sex).

Blood samples were taken from the left ventricle in 6 h light cycle fasted terminally anesthetized mice (9 months of age; n = 5–9/genotype/sex) (phenobarbital sodium (Dolethal); Vétoquinol, UK). Insulin and leptin were assayed using a two-plex electrochemical luminescence microtiter plate immunoassay (MesoScale Discovery, Gaithersburg, MD, USA).

### 2.5 Insulin tolerance test

Mice (6–8 months of age; n = 5–9/genotype/sex) were fasted for 6 h during the light cycle. Blood was sampled from tail vein immediately prior to insulin (1.1 U/kg IP, males; 0.8 U/kg IP, females) bolus, and 15, 30, 45, 60 and 90 min following bolus administration. Blood glucose was analyzed using AlphaTrak glucometer (Chicago, IL, USA).

### 2.6 Statistics

Data were analyzed with One-way, Two-way or Repeated Measures ANOVA or ANCOVA followed by Tukey's or Bonferroni *post hoc* tests. General linear models were also performed for energy expenditure analysis. For all analyses, significance was assigned at *P* < 0.05. Data are presented as mean ± SEM.

## 3 Results and discussion

### 3.1 Generation of *5-HT*_*2C*_*R*^*CRE*^ line

To clarify the source of POMC peptides that underpin energy balance and body weight regulation, we utilized a Cre recombinase-dependent and ARC specific reactivatable *Pomc*^*NEO*^ line [13] to restore *Pomc* synthesis within the discrete subset of cells expressing 5-HT_2C_Rs. To achieve this, we first generated a *5-HT*_*2C*_*R*^*CRE*^ line. To confirm that *5-HT*_*2C*_*R*^*CRE*^ is contained in cells expressing endogenous 5-HT_2C_R, *5-HT*_*2C*_*R*^*CRE*^ mice were intercrossed with B6.129X1-*Gt(ROSA)26Sortm1(EYFP)Cos*/J (*Rosa26*^*YFP*^) mice, which have a *loxP*-flanked STOP sequence followed by an *YFP* gene inserted into the Gt(ROSA)26Sor locus. Intercrossing with *5-HT*_*2C*_*R*^*CRE*^ mice removes the STOP sequence and YFP is visualized in *5-HT*_*2C*_*R*^*CRE*^ expressing cells (Figure S1A). Performing immunofluorescent staining in the ARC for YFP-immunoreactivity (IR) and fluorescent *in situ* hybridization (FISH) to label endogenous *5-HT*_*2C*_*R* mRNA revealed that the majority of Cre containing cells expressed endogenous *5-HT*_*2C*_*R* mRNA (Figure S1B).

### 3.2 Anatomical localization and genetic manipulation of subset of hypothalamic *Pomc* co-expressing 5-HT_2C_Rs

**Figure 1:**
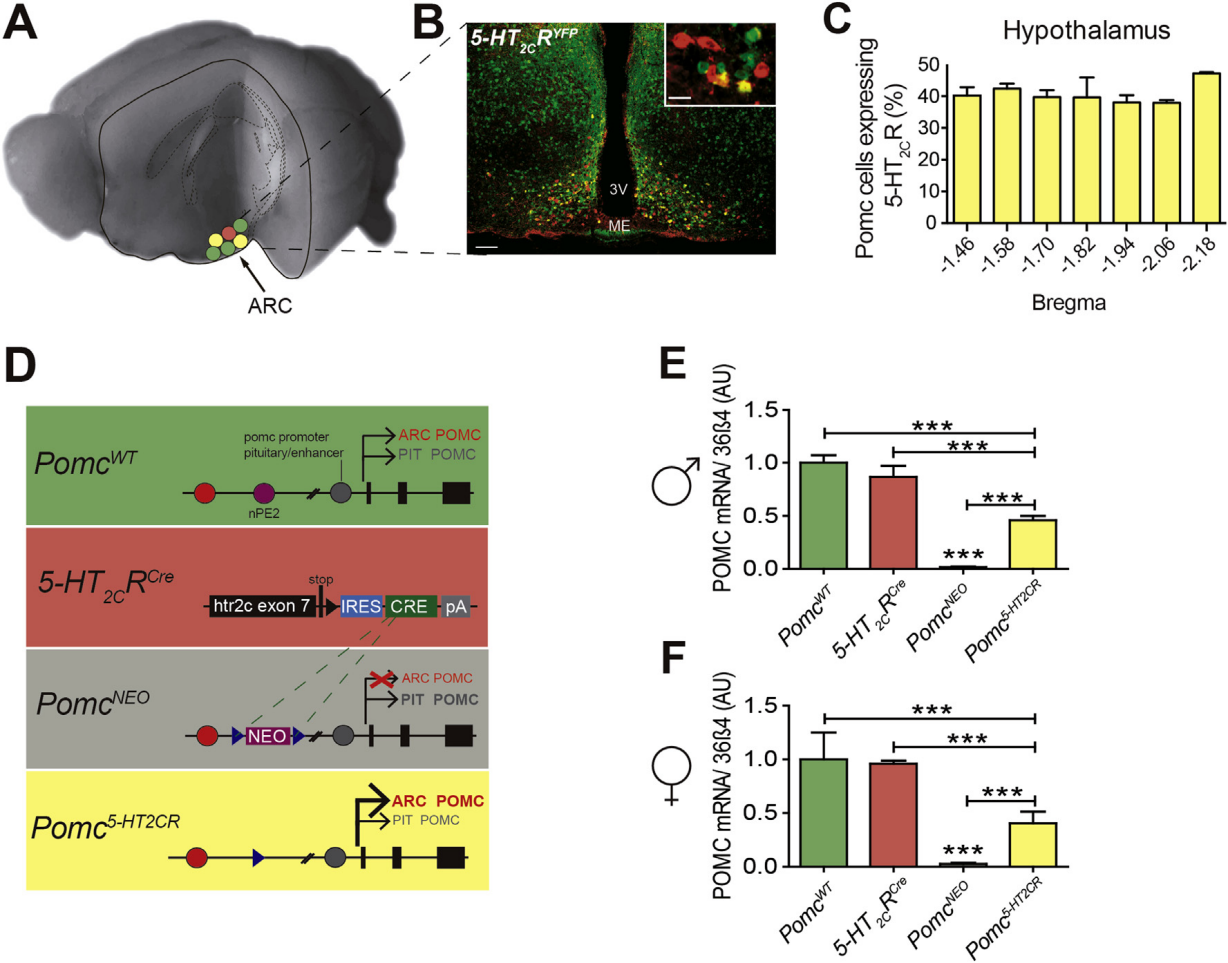
**Generation of mice with restored ARC *Pomc* function in 5-HT**_**2C**_**R expressing cells**. **(A,B)** ARC POMC neurons (POMC-IR, red) co-express 5-HT_2C_Rs (GFP-IR, green; co-labeled, yellow). Scale bar, 50 μm (inset) and 100 μm. **(C)** POMC-IR and 5-HT_2C_R (GFP-IR) co-expression by ARC bregma level. **(D)** Schematic of wild-type allele (*Pomc*^*WT*^) containing both neuronal *Pomc* enhancers, nPE1 and nPE2 (green); 5-HT_2C_R^CRE^ inserted after *ht2rc* exon 7 (red); a disrupted *Pomc*^*NEO*^ knockout allele carrying nPE2 deletion and a loxP-flanked-mediated disruption of nPE1 transcriptional activation function (gray); and a re-activated Pomc/5-HT_2C_ allele (*Pomc*^*5−HT2CR*^) (yellow). **(E)***Pomc* re-expression in 5-HT_2C_R neurons in male (F_3,19_ = 22.40, *P* < 0.0001) and **(F)** female mice (F_3,15_ = 18.40, *P* < 0.0001) normalized to *36β4* mRNA, relative to *Pomc*^*WT*^, in arbitrary units (AU). *P < 0.05, **P < 0.01, ***P < 0.001 as indicated.

To determine the anatomical localization of the subset of ARC POMC neurons specifically co-expressing 5-HT_2C_Rs, dual-immunofluorescent analysis was performed for GFP-IR and POMC-IR in the *5-HT*_*2C*_*R*^*YPF*^ line. This analysis revealed that approximately 40% of ARC POMC neurons co-express 5-HT_2C_Rs in male and female mice (Figure 1A–C). This co-expression profile is similar to that previously observed in rats [9]. Further analysis of anatomical co-localization indicated that this 40% co-expression rate was consistent across the rostral-caudal extent of the ARC (Figure 1C).

To investigate the physiological importance of this specific source of POMC peptides in the regulation of energy balance and body weight, we intercrossed a Cre recombinase-dependent and ARC specific reactivatable *Pomc*^*NEO*^ line to restore *Pomc* expression only within cells expressing 5-HT_2C_Rs (Figure 1D). As expected, *Pomc*^*NEO*^ mice had no detectable hypothalamic *Pomc* mRNA, whereas *Pomc* was restored by 44% in male and 41% in female *Pomc*^*5-HT2CR*^ mice (Figure 1E,F). This *Pomc* reactivation level is consistent with the anatomical co-localization determined above. No differences in *Pomc* expression among genotypes were detected in the brainstem, which includes the nucleus tractus solitarius (Figure S1C). Therefore, we created a genetic means to probe the specific function of a subset of ARC POMC neurons in the regulation of whole body energy balance and body weight.

### 3.3 Hypothalamic *Pomc* expressed exclusively within 5-HT_2C_R containing cells modulates energy intake in male and female mice

**Figure 2:**
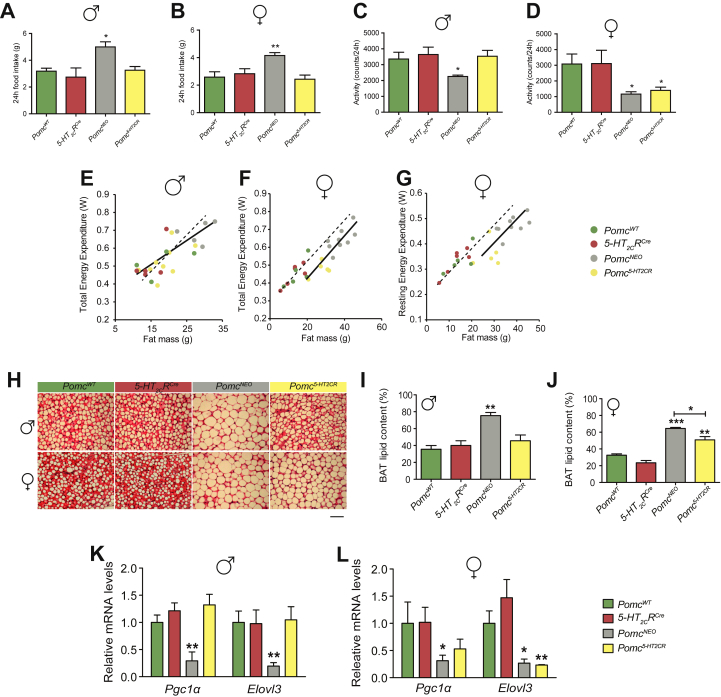
**Subpopulation of *Pomc* differentially modulates physical activity and energy expenditure in male and female mice**. 24 h food intake was significantly elevated in **(A)** male (F_3,12_ = 13.98, *P* < 0.001) and **(B)** female (F_3,11_ = 6.55, *P* < 0.001) ARC *Pomc* null (*Pomc*^*NEO*^) mice, and this was normalized by restoration of Pomc in 5-HT_2C_R neurons in *Pomc*^*5−HT2CR*^ mice. 24 h locomotor activity was significantly reduced in **(C)** male (F_3,17_ = 3.68, *P* < 0.05) and **(D)** female (F_3,18_ = 5.36, *P* < 0.05) *Pomc*^*NEO*^ mice, and this was normalized in male, but not female, *Pomc*^*5-HT2CR*^ mice. General linear model in **(E)** male and **(F)** female mice illustrating reduced total daily energy expenditure in *Pomc*^*NEO*^ and *Pomc*^*5-HT2CR*^ mice (solid line) compared to control siblings (dashed line) in females (group effect *P* < 0.01) but not in males (group effect *P* > 0.05). **(G)** General linear model revealing *Pomc*^*NEO*^ and *Pomc*^*5-HT2CR*^ females (solid line) display reduced resting metabolic rate compared to control siblings (dashed line) (group effect *P* < 0.01). **(H,I)** Lipid accumulation in interscapular brown adipose tissue (BAT; F_3,17_ = 11.61, *P* < 0.001) and relative expression of mitochondrial genes important for BAT thermogenesis, **(K)***Pgc-1α* (F_3,18_ = 8.638, *P* < 0.001) and *Elovl3* (F_3,18_ = 4.147, *P* < 0.05), were fully normalized by restoration of *Pomc* in 5-HT_2C_R neurons (*Pomc*^*5-HT2CR*^) in male mice. By contrast, *Pomc*^*5-HT2CR*^ female mice display an increase in **(H,J)** BAT lipid content (F_3,10_ = 40.00, *P* < 0.0001) and a downregulation in **(L)***Pgc-1α*(F_3,12_ = 8.941, *P* < 0.01) and *Elovl3* (F_3,18_ = 8.941, *P* < 0.01) compared to littermate controls. Data expressed as arbitrary units and expression of target genes corrected to the geometric average of four housekeeping genes:*18s*, *36β4*, *Gapdh* and *βactin*. Scale bar, 2000 μm *P < 0.05, **P < 0.01, ***P < 0.001 compared to all other genotypes, except in D, J and L compared to *Pomc*^*WT*^ and *5-HT*_*2C*_*R*^*Cre*^ mice.

Given that current obesity medication 5-HT_2C_R agonist lorcaserin improves obesity by influencing appetite, but not energy expenditure [19], and that 5-HT_2C_R agonists reduce food intake via increased activity of POMC neurons [9], [20], we surmised that POMC peptides synthesized exclusively in neurons expressing 5-HT_2C_Rs perform an essential role in the regulation of energy intake. To investigate this, we compared 24-hour food intake in *Pomc*^*NEO*^, *5-HT*_*2C*_*R*^*CRE*^, wild type and mice with restored *Pomc* expression only in cells expressing 5-HT_2C_Rs (*Pomc*^*5-HT2CR*^). As expected, male and female *Pomc*^*NEO*^ mice exhibited significant hyperphagia (Figure 2A,B; Figure S2A–C, S2G-I), consistent with previous reports [13]. Restoration of hypothalamic *Pomc* expression only within 5-HT_2C_R cells normalized 24-h food intake in both male and female mice (Figure 2A,B). Additional circadian analysis revealed that *Pomc*^*NEO*^ energy intake was significantly higher during the dark cycle in male (Figure S2B,C) and female (Figure S2H,I) mice, and this was normalized by the restoration of *Pomc* expression in 5-HT_2C_R cells in both sexes. Next, we tracked 24-h food intake in male and female mice by genotype until 6 months of age. We observed that *Pomc*^*NEO*^ hyperphagia persisted with age, whereas *Pomc*^*5-HT2CR*^ mice continued to consume food at a level consistent with control *5-HT*_*2C*_*R*^*CRE*^ and wild type littermates (Figure S2A,G). Consequently, these data indicate that POMC peptides synthesized exclusively in neurons expressing 5-HT_2C_Rs are sufficient to mediate POMC's effects on food intake.

### 3.4 Unexpected sex difference in physical activity and energy expenditure modulated by hypothalamic *Pomc* exclusively expressed within 5-HT_2C_R containing cells

Analysis of energy homeostasis in *Pomc*^*5-HT2CR*^ mice uncovered a substantial and unexpected sex difference in the molecular regulation of physical activity and energy expenditure. In both males and females, *Pomc*^*NEO*^ mice exhibited significantly reduced 24-h locomotor activity, which was accounted for by a reduction in activity within the dark, but not light cycle (Figure 2C,D; Figure S2E,F,K,L). However, restoration of *Pomc*^*5-HT2CR*^ function fully normalized physical activity only in male mice (Figure 2C; Figure S2E,F). While a significant effect of body fat mass on total daily energy expenditure was detected in males (Figure 2E), this did not vary by genotype. Conversely, in female mice, restoration of *Pomc*^*5-HT2CR*^ function had no impact on reduced physical activity (Figure 2D, Figure S2K,L) or total daily energy expenditure (Figure 2F). Thus, in females, a significant genotype effect of fat mass on total daily energy expenditure was observed. A general linear model using both fat and lean mass explained 80% of the variance in energy expenditure in pooled female wild type and *5-HT*_*2C*_*R*^*CRE*^ mice. Both *Pomc*^*5-HT2CR*^ and *Pomc*^*NEO*^ female mice displayed significantly reduced total daily energy expenditure compared to wild type and *5-HT*_*2C*_*R*^*CRE*^ female siblings (Tukey's *post hoc P* < 0.01) but did not differ from each other (*P* > 0.05). We next explored the impact of genotype and body composition on resting metabolic rate in female mice (Figure 2G). A general linear model using both fat and lean mass explained 91.8% of the variance in energy expenditure in pooled female wild type and *5-HT*_*2C*_*R*^*CRE*^ mice. These results reveal that both *Pomc*^*NEO*^ and *Pomc*^*5-HT2CR*^ female mice had significantly reduced resting energy expenditure compared to wild type and *5-HT*_*2C*_*R*^*CRE*^ siblings (Tukey's *post hoc P* < 0.01) but did not differ from each other (*P* > 0.05).

Brown adipocytes contain a large number of mitochondria for thermogenesis, and nutrient oxidation in BAT can account for up to 60% of the total energy expenditure of mice [21], [22]. Melanocortinergic regulation of BAT thermogenesis and energy expenditure has been reported via Mc4rs expressed by cholinergic preganglionic sympathetic neurons within the intermediolateral nucleus of the thoracic spinal cord (IML) [23], [24], [25]. The IML is directly innervated by ARC POMC neurons [26], [27] and postganglionic neurons innervating brown adipose tissue receive projections from the IML [28], [29], [30], suggesting a pathway through which POMC neurons may participate in the regulation of BAT thermogenesis and energy expenditure.

We therefore next examined whether *Pomc* exclusively synthesized within 5-HT_2C_R expressing neurons influences BAT function. In male and female mice, genetic inactivation of ARC *Pomc* was associated with increased lipid accumulation in BAT (Figure 2H–J) and reduced expression of *Pgc-1a* and *Elolv3*, mitochondrial genes important for BAT thermogenesis (Figure 2K–L). In male mice, restoration of *Pomc*^*5-HT2CR*^ function normalized BAT lipid accumulation (Figure 2H,I) and *Pgc-1a* and *Elolv3* expression (Figure 2K). In contrast, *Pomc*^*5-HT2CR*^ female mice displayed an increase in BAT lipid content (Figure 2H,J) and a downregulation in thermogenic gene expression (Figure 2L) compared to control littermates. These data suggest that impaired BAT thermogenesis in female *Pomc*^*NEO*^ and *Pomc*^*5-HT2CR*^ mice contributes to the observed reduction in whole body energy expenditure.

Taken together, these data signify that ARC POMC regulates both physical activity related energy demands and resting metabolism and that restoration of POMC function within the subset of 5-HT_2C_R expressing neurons is not sufficient to appropriately regulate energy expenditure or BAT function in female mice. No differences in respiratory exchange ratio (RER) were detected by genotype in either sex, and, in the combined data set, there was no sex effect on the RER (Figure S2D,J). However, we uncovered an unexpected sex difference in the molecular underpinnings driving physical activity and determined that *Pomc*^*5-HT2CR*^ have a broader, sex-specific, function in the regulation of energy usage.

### 3.5 Unexpected sex difference in body weight regulation modulated by hypothalamic *Pomc* exclusively expressed within 5-HT_2C_R containing cells

**Figure 3:**
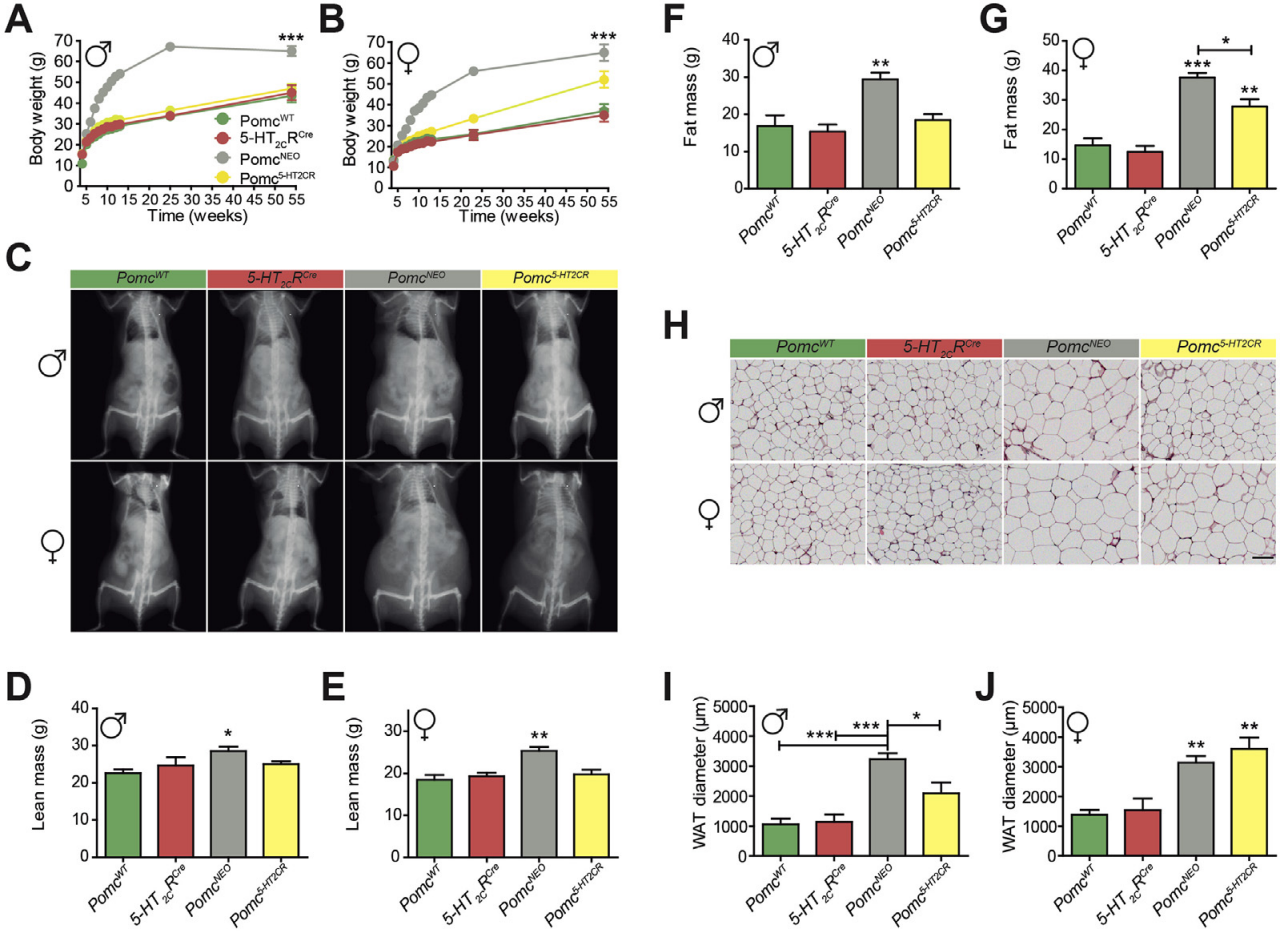
**Subpopulation of *Pomc* differentially modulates body weight and adiposity in male and female mice**. **(A,B)** Body weight (male, F_3,20_ = 114.35, *P* < 0.001; female F_3,19_ = 60.80, *P* < 0.001), **(C, F, G)** fat mass (male F_3,20_ = 9.43, *P* < 0.001; female F_3,22_ = 42.04, *P* < 0.001), **(H–J)** gonadal white adipocyte diameter (male F_3,14_ = 13.98, *P* < 0.001; female F_3,13_ = 11.87, *P* < 0.001) were increased in ARC *Pomc* null (*Pomc*^NEO^) mice and normalized by restoration of *Pomc* in 5-HT_2C_R neurons (*Pomc*^*5-HT2CR*^) in male but not female mice. However, female *Pomc*^*NEO*^ mice still exhibited significantly greater body weight and fat mass, but not adipocyte size, compared with *Pomc*^*5-HT2CR*^ mice. **(D, E)** Both male and female *Pomc*^*NEO*^ mice exhibited significantly greater lean mass compared to *Pomc*^*5-HT2CR*^ mice and controls. Scale bar, 2000 μm *P < 0.05, **P < 0.01, ***P < 0.001 compared to control *Pomc*^*WT*^ and *5-HT*_*2C*_*R*^*CRE*^ siblings, except where noted in D, E, G and I.

We next investigated the ramifications of this discrete source of ARC POMC peptides on body weight and adiposity. Consistent with the metabolic consequence of restored energy balance in male *Pomc*^*5-HT2CR*^ mice, a prevention of obesity in male *Pomc*^*5-HT2CR*^ mice was observed compared to *Pomc*^*NEO*^ littermates (Figure 3A). Furthermore, male *Pomc*^*5-HT2CR*^ mice displayed levels of lean mass (Figure 3C,D), fat mass (Figure 3F) and leptin levels (Figure S3A) that were comparable to control *5-HT*_*2C*_*R*^*CRE*^ and wild type littermates. Likewise, male *Pomc*^*NEO*^ mice had larger white adipocytes and this phenotype was normalized in male *Pomc*^*5-HT2CR*^ mice (Figure 3H,I).

In contrast, ARC *Pomc* synthesized within neurons expressing 5-HT_2C_Rs was not sufficient to regulate adiposity in female mice. Female *Pomc*^*5-HT2CR*^ mice were genetically predisposed to develop obesity compared to wild type and *5-HT*_*2C*_*R*^*CRE*^ female siblings (Figure 3B), with increased fat mass (Figure 3C,G) and displayed significantly larger white adipocytes (Figure 3H,J). This elevation in body weight and fat mass was less severe than that produced by full ARC *Pomc* nulls, though both *Pomc*^*NEO*^ and *Pomc*^*5-HT2CR*^ mice showed comparable increases in white adipocyte size. However, only *Pomc*^*NEO*^ mice exhibited statistically increased lean mass (Figure 3E) and leptin levels (Figure S3B) and this was corrected in *Pomc*^*5-HT2CR*^ mice. These data reveal that the subpopulation of ARC POMC expressed within 5-HT_2C_R containing neurons is sufficient to regulate whole body energy balance, body weight and adiposity in male, but not female, mice.

### 3.6 Hypothalamic *Pomc* expressed exclusively within 5-HT_2C_R containing cells modulates insulin sensitivity in male and female mice

**Figure 4:**
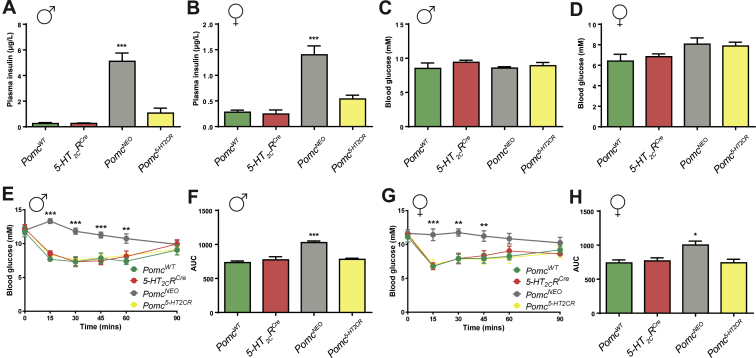
**Subpopulation of *Pomc* modulates insulin sensitivity in male and female mice**. **(A,B)** Plasma insulin (male, F_3,21_ = 63.20, *P* < 0.0001; female F_3,23_ = 20.33, *P* < 0.0001) was increased in ARC *Pomc* null (*Pomc*^NEO^) mice and normalized by restoration of *Pomc* in 5-HT_2C_R neurons (*Pomc*^5-HT2CR^). **(C,D)** Fasting blood glucose was not statistically different among genotypes (male, F_3,15_ = 0.70, *P* > 0.05; female F_3,18_ = 2.30, *P* > 0.05). Both male **(E,F)** and female **(G,H)***Pomc*^NEO^ mice exhibited impaired responses in an insulin tolerance test compared with control littermates, which were normalized by restoration of *Pomc* in 5-HT_2C_R neurons (*Pomc*^5-HT2CR^); **(E,G)** Insulin tolerance tests (male, F_3,104_ = 33.09, *P* < 0.0001; female F_3,102_ = 24.39, *P* < 0.0001) and **(F,H)** their respective areas under the curve (AUC) (male, F_3,18_ = 18.06, *P* < 0.0001; female F_3,17_ = 6.56, *P* < 0.01). *P < 0.05, **P < 0.01, ***P < 0.001 compared to all other genotypes.

Recent reports revealed that though *Pomc*^*NEO*^ mice exhibit insulin resistance, they display normal glucose levels and improved glucose tolerance, primarily by increasing glycosuria [31], [32]. We next considered whether *Pomc* synthesized exclusively within 5-HT_2C_R expressing cells is sufficient to normalize *Pomc*^*NEO*^ hyperinsulinemia and impaired insulin tolerance. As expected, severely hyperphagic and obese male and female *Pomc*^*NEO*^ mice exhibited pronounced hyperinsulinemia (Figure 4A,B) with normal fasting blood glucose (Figure 4C,D), a pattern indicating insulin resistance compensated by increased insulin secretion from pancreatic beta cells. Consistent with the metabolic consequence of normalized energy balance and adiposity in male *Pomc*^*5-HT2CR*^ mice, hyperinsulinemia was corrected in male *Pomc*^*5-HT2CR*^ mice compared to *Pomc*^*NEO*^ littermates (Figure 4A). Despite disrupted energy balance and pronounced obesity, female *Pomc*^*5-HT2CR*^ mice also displayed insulin levels comparable to wild type and *5-HT*_*2C*_*R*^*CRE*^ siblings (Figure 4B). Examining insulin sensitivity further, we found that both male (Figure 4E,F) and female (Figure 4G,H) *Pomc*^*NEO*^ mice exhibited impaired responses in an insulin tolerance test compared with control littermates, and this was normalized by restoring *Pomc* within 5-HT_2C_R expressing cells. Taken together, these results indicate that despite a sex difference in energy balance and obesity, *Pomc* synthesized in 5-HT_2C_R expressing cells is sufficient to mediate POMC's effects on insulin sensitivity in both male and female mice.

These data reveal a discrete and specific sex difference in the regulation of body weight, fat accumulation and adipocyte size driven by the subpopulation of hypothalamic POMC peptides exclusively synthesized in neurons expressing 5-HT_2C_Rs. Interestingly, POMC within the subpopulation of ARC leptin receptor expressing neurons was sufficient to normalize energy balance and body weight in both male and female *Pomc*^*NEO*^ mice [32]. Thus, these data reveal a specific and functionally distinct role of POMC within the subset of cells examined here, providing further support for the functional heterogeneity of ARC derived POMC peptides.

The sex difference in energy storage is consistent with significantly reduced energy usage in female *Pomc*^*5-HT2CR*^ mice. Given that food intake is normalized in female *Pomc*^*5-HT2CR*^ mice, this genetic model reflects a new example of the cumulative impact of reduced physical activity and energy expenditure on body weight and adiposity with time. It may be noted that in the *Pomc*^*NEO*^ mice, the impact on energy expenditure was much greater in the females compared to males (compare Figure 2E,F) and the corresponding fat mass also much greater (compare Figure 3F–G). Therefore, the localized synthesis of POMC peptides within a subset of neurons within the discrete brain region the ARC produces a substantial difference in the magnitude of adiposity accumulation in male and female mice. This phenotype cannot simply be explained by impairing POMC activity by estrogens, because the inactivation of estrogen receptor specifically within ARC POMC neurons does not impact fat mass or reduce energy expenditure [33].

## 4 Conclusions

These findings support the functional heterogeneity of ARC POMC, revealing that the source synthesized within 5-HT_2C_R expressing neurons is sufficient to regulate energy intake and insulin sensitivity in male and female mice. Moreover, these data provide evidence for a specific neurochemical basis for levels of reduced physical activity and reveal that the molecular underpinnings of the impetus to engage in physical activity are differentially modulated in males and females. However, physical activity comprises only one means of utilizing energy and our data further show that the same neuronal population plays a key role, modulated by sex, in the regulation of resting rates of expenditure. Consequently, these data uncovered an unexpected sex difference, mediated by POMC, in total energy expenditure, thermogenic activity of BAT and adiposity. These findings provide evidence that males and females are hardwired differently in their regulation of energy balance. Given the reported reduction of POMC neuron activity in middle age in mice [7], these data may have translational relevance by providing a potential molecular explanation for the global sex differences in obesity prevalence. Finally, these data may have broad implications for future sex-specific strategies in treating overweight and obesity.

## Figures and Tables

**Figure:**
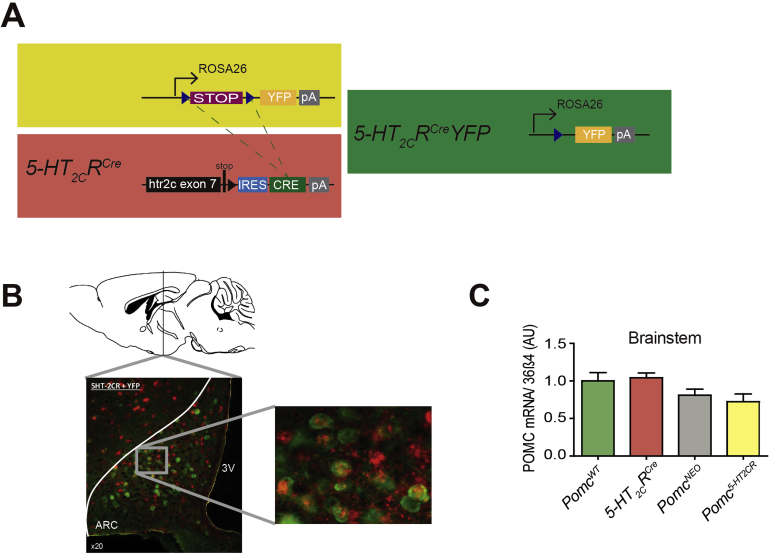


**Figure:**
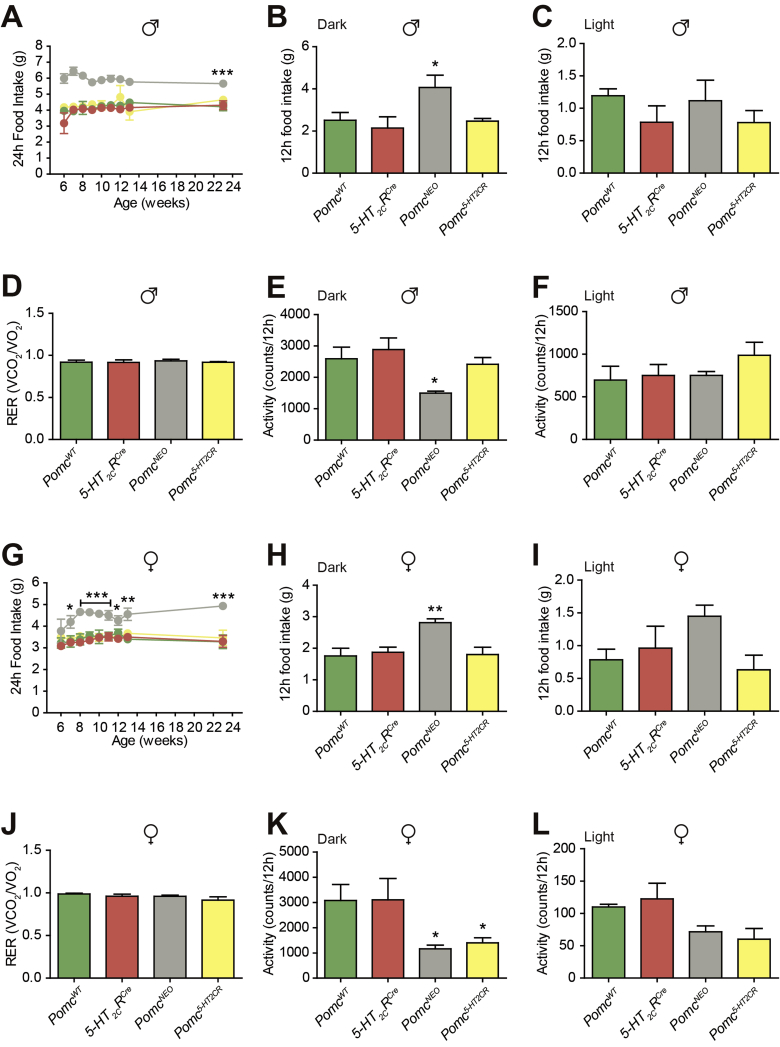
2

**Figure:**
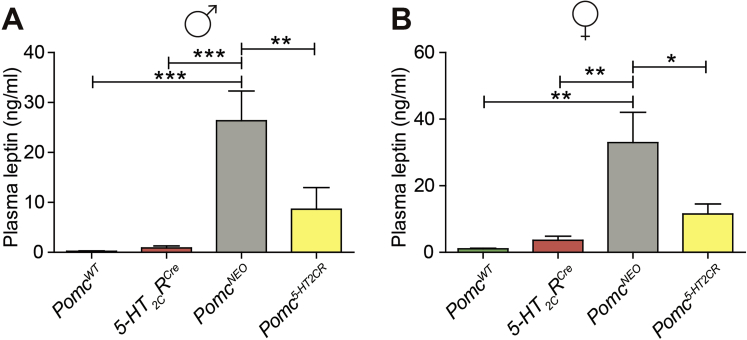
3
