# Supplementary material for: Sex difference in physical activity, energy expenditure and obesity driven by a subpopulation of hypothalamic POMC neurons
Source: Mol Metab. 2016 Jan 22;5(3):245–52. doi: 10.1016/j.molmet.2016.01.005 (PMC4770275; doi:10.1016/j.molmet.2016.01.005)
Supplement: Supplementary file 1 [file mmc1.doc]

**METHODS**

***Dual Fluorescence in situ hybridization (FISH) with IHC***

An RNA expression vector (pBluescript SK-) containing the 3-kilobase (kb) coding region of the 5-HT_2C_R cDNA was used to generate single-stranded RNA probes . Briefly, *in vitro* transcription was performed using Digoxigenin (DIG)-RNA Labelling Mix (Roche, Mannheim, Germany). Sections were treated with 1% Sodium Borohydride solution and 0.25% Acetic Anhydride in Triethanolamine (TEA) solution. Sections were then incubated in a hybridization buffer containing a dioxigenin (DIG)-UTP-labelled 5-HT_2C_R riboprobe overnight at 55°C. Sections were rinsed in a standard sodium citrate/50% Formamide solution, rinsed in an RNase (0.02mg/ml RNase A) solution, incubated in 3% H_2_O_2_ solution for 30 min and blocked in 2% Sheep serum (Sigma, Saint Louis, USA). Sections were then incubated with Anti-DIG antibody (1:5000, Roche, Mannheim, Germany), treated with TSA PLUS Biotin Kit (Perkin Elmer, Waltham, USA ) and revealed with Streptavidin conjugated Alexa Fluor® 568 (1/2000, Lifetechnologies, Carlsbad, USA). After FISH procedure, sections were blocked again and incubated with anti-GFP primary antibody (1/1000, Abcam, Cambridge, UK) overnight at 4°C using IHC protocol described above. The sections were incubated with Alexa Fluor® 488 Donkey Anti-Goat secondary antibody (1/500, Invitrogen^TM^ Lifetechnologies, Carlsbad, USA) for 1h. Tissue was then mounted on slides, coverslipped and single- and dual-labeling assessed in the ARC.

***Metabolic profile***

Body weight was measured from weaning up to 1 year of age. Home cage 24 hour food intake was measured up to 6 months of age. At 9 months of age, a more detailed energy balance profile was performed, including light and dark cycle food intake, locomotor activity and energy expenditure assessment using indirect calorimetry in a Metabolic-Trace (Meta-Trace) system (Ideas Studio, UK). Body composition was also analyzed at 7-9 months of age using dual-energy x-ray absorptiometry (DEXA) Lunar PIXImus2 mouse densitometer (General Electric Medical Systems, Fitchburg, WI, USA).

Total daily and resting energy expenditure data was determined using ANCOVA . Briefly we entered genotype as a fixed factor and both fat and lean mass as covariates along with all 2-way and three-way interactions in a general linear model. Non-significant interaction terms were removed and the model re-run. Fat mass and lean mass were highly correlated predictors and because fat mass was more variable it entered the model in preference to lean mass. When a genotype effect was found we located the effect by running a regression model to generate a predictive equation using the wild type mice and *5-HT_2C_R^Cre^* mice. We then used this predictive equation to generate predicted metabolic rates for all animals and compared the predictions to the observed metabolic rates. We used this approach because there was no overlap in the body and fat masses of some groups, and it is well known that ANCOVA does not cope well with locating group effects in that situation . The analysis was run separately for males and females. A similar approach was used to analyse the respiratory exchange ratio (RER).

**Supplementary Table 1. Real time PCR Oligonucleotide Sequences**

| Target Gene | Forward Primer | Reverse Primer | Probe |
| --- | --- | --- | --- |
| Mouse pGC1a | AAC CAC ACC CAC AGG ATC AGA | CTC TTC GCT TTA TTG CTC CAT GA | CAA ACC CTG CCA TTG TTA AGA CCG AGA A |
| Mouse Elovl6 | TGC AGG AAA ACT GGA AGA AGT CT | ATG CCG ACC ACC AAA GAT AAA |  |
| 18S | CGG CTA CCA CAT CCA AGG AA | GCT GGA ATT ACC GCG GCT |  |
| 36b4 | AGA TGC AGC AGA TCC GCA T | GTT CTT GCC CAT CAG CAC C |  |
| βactin | GCT CTG GCT CCT AGC ACC AT | GCC ACC GAT CCA CAC AGA GT | ATC AAG ATC ATT GCT CCT CCT GAG CGC |

**Supplementary Figure Legends**

**Figure S1**. **Generation of *5-HT_2C_R^YFP^* line. (A)** *5-HT_2C_R^Cre^* line was intercrossed with a B6.129X1-*Gt(ROSA)26Sortm1(EYFP)Cos*/J (*Rosa26^YFP^*, Jackson Labs), which has a *loxP*-flanked STOP sequence followed by an Enhanced Yellow Fluorescent Protein (YFP) gene inserted into the Gt(ROSA)26Sor locus. Intercrossing with *5-HT_2C_R^Cre^* mice removes the STOP sequence and YFP is visualized in *5-HT_2C_R^Cre^* expressing cells. **(B)** Mouse brain with coronal section to illustrate the ARC validation of *5-HT_2C_R^YFP^* mice showing dual-fluorescent YFP-immunoreactivity (green) and *5-HT_2C_R* mRNA (red) using fluorescent *in situ* hybridization (FISH) in male and female mice (n=6). As expected, YFP cells expressed *5-HT_2C_R* mRNA (red overlaying green), illustrating that Cre is expressed in endogenous *5-HT_2C_R* mRNA containing cells. **(C)** No differences in *Pomc* expression were detected by genotype in male or female mice in the nucleus of the solitary tract (NTS) normalized to 36βB4 rRNA, relative to *Pomc^WT^*, in arbitrary units (AU) demonstrating that *Pomc^NEO^* specifically prevents *Pomc* transcription in the ARC, not NTS. Data are presented as mean±SEM of 8-13 mice per genotype.

**Figure S2**. **Phenotypic characterization of energy balance in male and female mice.** 24h food intake was significantly elevated in **(A)** male (F_3,12_=13.988, *P*<0.001) and **(G)** female (F_3,11_=6.55, *P<*0.001) *Pomc^NEO^* mice over 6 months; and this was normalized to that of control siblings in *Pomc^5-HT2CR^* mice, due to normalized food intake during the dark-cycle in both (**B**) males (F_3,17_=5.40, *P<*0.01) and (**H)** females (F_3, 18_=8.83, *P<*0.001). (**C, I**) Food intake and (**F, L**) locomotor activity did not vary by genotype within the light cycle, when mice are typically less active. (**D, J**) Average 24h RER also did not vary by sex or genotype. However, a significant sex-difference by genotype was observed in dark-cycle physical activity. (**E**) Both male (F_3,17_=5.872, *P*<0.01) and **(K)** female (F_3,19_=5.050, *P*<0.01) *Pomc^NEO^* mice were significantly less active during the dark cycle compared to their wild-type and *5-HT_2C_R^Cre^* littermates. Restoration of *Pomc* within 5-HT_2C_R ARC cells reversed the hypolocomotor phenotype of male but not female *Pomc^5-HT2CR^* mice. Data are presented as mean±SEM of 4-9 mice per genotype. **P<*0.05, ***P<*0.01, ****P<*0.001 compared to all other genotypes except K **P*<0.01 compared to control *Pomc^WT^* and *5-HT_2C_R^Cre^* mice as assessed by one-way ANOVA followed by Tukey's test.

**Figure S3. Plasma leptin in male and female mice.** Plasma leptin levels were significantly higher in (**A**) male (F_3, 21_=18.51, *P*<0.001) and (**B**) female (F_3,23_=6.038, *P*<0.01) *Pomc^NEO^* mice. Restoration of *Pomc* expression in *Pomc^5-HT2CR^* corrected plasma leptin in both male and female mice to a level statistically similar to control mice. **P<*0.05, ***P<*0.01, ****P<*0.01

**Supplementary References**

1. Julius D, MacDermott AB, Axel R, Jessell TM. Molecular characterization of a functional cDNA encoding the serotonin 1c receptor. Science. 1988 Jul;241(4865):558-64.

2. Molineaux SM, Jessell TM, Axel R, Julius D. 5-HT1c receptor is a prominent serotonin receptor subtype in the central nervous system. Proc Natl Acad Sci U S A. 1989 Sep;86(17):6793-7.

3. Tschöp MH, Speakman JR, Arch JR, Auwerx J, Brüning JC, Chan L, et al. A guide to analysis of mouse energy metabolism. Nat Methods. 2012 Jan;9(1):57-63.

4. Speakman JR. Measuring energy metabolism in the mouse - theoretical, practical, and analytical considerations. Front Physiol. 2013;4:34.
